# Efficiency and Safety of Dental Implantation in the Area of Hyperdense Jaw Lesions: A Narrative Review

**DOI:** 10.3390/dj10060107

**Published:** 2022-06-10

**Authors:** Kimya Taghsimi, Andrey Vyacheslavovich Vasilyev, Valeriya Sergeevna Kuznetsova, Angelina Vladimirovna Galtsova, Varditer Agabekovna Badalyan, Igor Ivanovich Babichenko

**Affiliations:** 1 Department of Pathological Anatomy, Peoples’ Friendship University of Russia (RUDN University), 6 Miklukho-Maklaya St., 117198 Moscow, Russia; vav-stom@yandex.ru (A.V.V.); daffabilis@yandex.ru (A.V.G.); babichenko-ii@rudn.ru (I.I.B.); 2 Central Research Institute of Dental and Maxillofacial Surgery, Timur Frunze St., 16, 119021 Moscow, Russia; tilia7@yandex.ru (V.S.K.); krisdent@mail.ru (V.A.B.); 3 Research Centre for Medical Genetics, Moskvorechye St., 1, 115478 Moscow, Russia; 4 Department of Dentistry of the Institute of Dentistry E.V. Borovsky, I.M. Sechenov First Moscow State Medical University of the Ministry of Health of the Russian Federation (Sechenov University), 119991 Moscow, Russia

**Keywords:** hyperdense lesion, dental implantation, osteoid osteoma, odontoma, osteoblastoma, cementoblastoma, cement-osseous dysplasia, idiopathic osteosclerosis, condensing osteitis

## Abstract

Background. Mineralized lesions of the jaws are often found incidentally on radiographs and computed tomography. Most of them are benign, and only a few rare cases are associated with malignant transformation. However, there is little clinical data on successful rehabilitation with implants in patients with mineralized lesions. This narrative review aimed to study the efficiency and safety of dental implantation in the area of hyperdense lesions. Materials and Methods. A PubMed, Google Scholar, and Science Direct database search was carried out with keywords and manually. Results. The literature exploration identified 323 articles; only 19 of them matched the search criteria and reported cases about dental implantation in the lesion area. It has been shown that in 84.2% of described cases, dental implantation was successful: in the osteoid osteoma, odontoma, cementoblastoma, idiopathic osteosclerosis, and condensing osteitis areas dental implantation was performed without any complications. The possibility of lesion recurrence and implant failure limited the use of dental implants in the area of osteoblastoma and cemento-osseous dysplasia. Although most cases of dental implantation in hyperdense jaw lesions were successful and were not accompanied by complications, further research is needed.

## 1. Introduction

Radiolucent lesions, such as periapical and follicular cysts and odontogenic tumors, widely described in the literature, are well diagnosed and have a clear treatment strategy, while radiopaque lesions of the jaw are less studied [1,2,3]. If they are located in areas that do not require therapeutic or surgical intervention, they remain incidental findings on radiographs and computed tomography [4]. If dental implants are necessary, a number of questions about the effectiveness and safety of implantation arise [5,6,7,8,9].

Impacted and ankylosed teeth, as well as residual roots are common and have a similar radiological appearance to some hyperdense lesions. There are some data on the clinical success of oral rehabilitation by implants placed in contact with dental tissues [10,11,12,13]. Histological studies have shown that cement is formed on the implant surface that is contacted with the root [14]. According to the hypothesis on implants placed through the dentin and pulp chamber, tertiary dentin, originating from the potential differentiation of pulp stem cells, can be formed [15].

Regarding hyperdense lesions, there are currently no clinical guidelines on dental implantation in these areas. It is related to the rarity of such lesions, the difficulties in diagnosis, problems in implant insertion, and complications during the operation and in the postoperative period [6,7,9]. In this regard, in the literature, only individual clinical cases are presented. In the studies, there are significant differences in the methods of lesion diagnosis, the tactics of surgical intervention, follow-up duration, and results [16,17,18]. Also, there are no reviews that could systematize data and evaluate the possibility of implantation in the area of hyperdense jaw lesions.

The purposes of this narrative review were systematization of information about hyperdense jaw lesions and summarizing research data on dental implantation in the area of these lesions. The review illustrates benign bone and cartilage tumors, such as osteoid osteoma, odontoma, and osteoblastoma, benign mesenchymal odontogenic tumors, such as cementoblastoma, and osteochondromatous lesions, such as cement-osseous dysplasia. In addition, lesions with similar manifestations–exostosis, idiopathic osteosclerosis, and condensing osteitis–are discussed.

## 2. Materials and Methods

A literature search was performed in the articles published from 1978 to January 2022 that included a combination of the terms “dental implantation“ and “neoplasms”/“radiopaque lesion”/“osteoid osteoma”/“odontoma”/“osteoblastoma”/“cementoblastoma”/“cemento-osseous dysplasia”/“exostosis”/“idiopathic osteosclerosis”/“condensing osteitis”. The inclusion criteria were: all types of articles, articles published in the PubMed, Google Scholar, and Science Direct databases, and related only to humans. The exclusion criteria were: articles that were not published in English, studies containing keywords but not relevant to the research topic, and articles that were not available in full text. From the articles retrieved in the first round of the search, additional references were identified by a manual search among the cited references.

## 3. Results

A total of 323 articles associated with dental implantation in the area of hyperdense lesions were found. Considering the exclusion criteria, 304 articles were excluded according to language limitations (not in English, *n* = 2), irrelevance to the main subject of the review (*n* = 300), non-full-text articles (*n* = 2). Nineteen articles were finally used (*n* = 19). All of them were case reports or case series: 2 for osteoid osteoma, 5 for odontoma, 0 for osteoblastoma, 2 for cementoblastoma, 7 for cemento-osseous dysplasia, 1 for condensing osteitis, and 2 for idiopathic osteosclerosis. Of these, 3 were devoted to implant failure in the FCOD area, which is 15.8% of the number of reported cases. Based on the data received, it can be considered that in 84.2% of cases, implantation in the area of hyperdense jaw lesions was successful. However, the results obtained are controversial, due to the abstract of the unified implantation protocol and the follow-up duration period.

### 3.1. Osteoid Osteoma

Osteoid osteoma is a rare benign bone tumor without infiltrative or metastatic potential. The lesions have been observed more in the mandible than in the maxilla, particularly in the angle of the mandible and in the area of the lingual aspect [19,20].The etiology of osteoid osteoma is unknown, but trauma, family history, developmental abnormalities, and inflammatory processes associated with systemic diseases, such as encephalocraniocutaneus lipomatosis (ECCL), are contributing factors [21,22].

Usually, osteoid osteoma is asymptomatic, but some articles report swelling and pain [23,24,25,26]. In some cases, it is accompanied by headache, recurrent sinusitis, paranasal sinus involvement, and ophthalmologic complaints.

#### 3.1.1. Histopathologic Features

Histologically, osteoid osteoma has two variants. The first is centrally located lamellar trabeculae of cancellous bone with large fibrofatty bone marrow surrounded by osteoblasts and scattered osteoclasts [27]. The second refers to dense, compact bone with sparse bone marrow tissue [22]. Trabeculae are thin and short but sclerotic and broad, rimmed by a single layer of osteoblasts. Cortical lesions that produce bone replacement stimulate the overlying periosteum, thereby establishing the new bone to a typical structure [28].

#### 3.1.2. Radiographic Features and Differential Diagnosis

Radiographically, osteoid osteoma is represented as a central radiopacity with well-circumscribed round-to-ovoid radiolucency or a nidus with a surrounded sclerotic zone. The size of the nidus is less than 1.5 cm. Sometimes superimposing other bony structures or obstructions due to surrounding dense sclerosis make the radiolucent nidus unrecognizable. In some cases, the absence of a radiolucent rim around the radiopaque mass can be observed [29,30,31].

Osteoid osteoma could be differentiated from osteoblastoma, osteomyelitis, odontoma, cementoblastoma, condensing osteitis, and cement-osseous dysplasia. Compared with osteoid osteoma, osteoblastomas grow rapidly and cause significant pain. It is more extensive, tends to be more aggressive, and can undergo a malignant transformation, while osteoid osteoma is small, benign, and self-limited. Histologically, both have similarities. The diagnosis is based on growth rate, nidus size, and presence of reactive bone formation. Lesions less than 1.5 cm are considered osteoid osteoma and lesions larger than 1.5 cm are osteoblastoma [32,33].

Osteomyelitis is prone to be another differential diagnosis of osteoid osteoma. Radiographically, acute osteomyelitis is represented as a change in a trabecular pattern similar to a spongy trabecular bone: this change is followed by loss of the lamina dura and extension of the periodontal ligament. As time passes, the recognition of boundaries becomes obscured, resulting in the transformation into chronic osteomyelitis [34]. In addition, there is an abscess in the oral cavity, which is accompanied by intermittent pain and a rapid increase in the size of more than 2 cm [35].

Osteomas may be confused radiographically with odontomas. Both odontoma and osteoma growth in the early stage is asymptomatic, but histologically, odontomas arise from disorganized differentiation of ameloblasts and odontoblasts related to constant pressure and trauma [36].

Cementoblastoma can be mistaken for an osteoma. However, in the case of cementoblastoma, the radiolucent rim surrounds the radiopacity and is connected or embedded in the tooth root. In addition, this lesion is accompanied by pain and swelling in the alveolar ridge area. Histologically, cementoblastoma consists of dense masses of acellular cementum-like material with basophilic reversal lines in a fibrous stroma.

Condensing osteitis has significant histological and radiographic similarities with osteoid osteoma, but it is mainly associated with periapical inflammatory disease that results from a reaction of the dental pulp. Additionally, osteoma, unlike condensing osteitis, osteoblastoma, and cementoblastoma, can be located in the toothless parts of the jaw, where implants are placed the most frequently.

**Figure 1:**
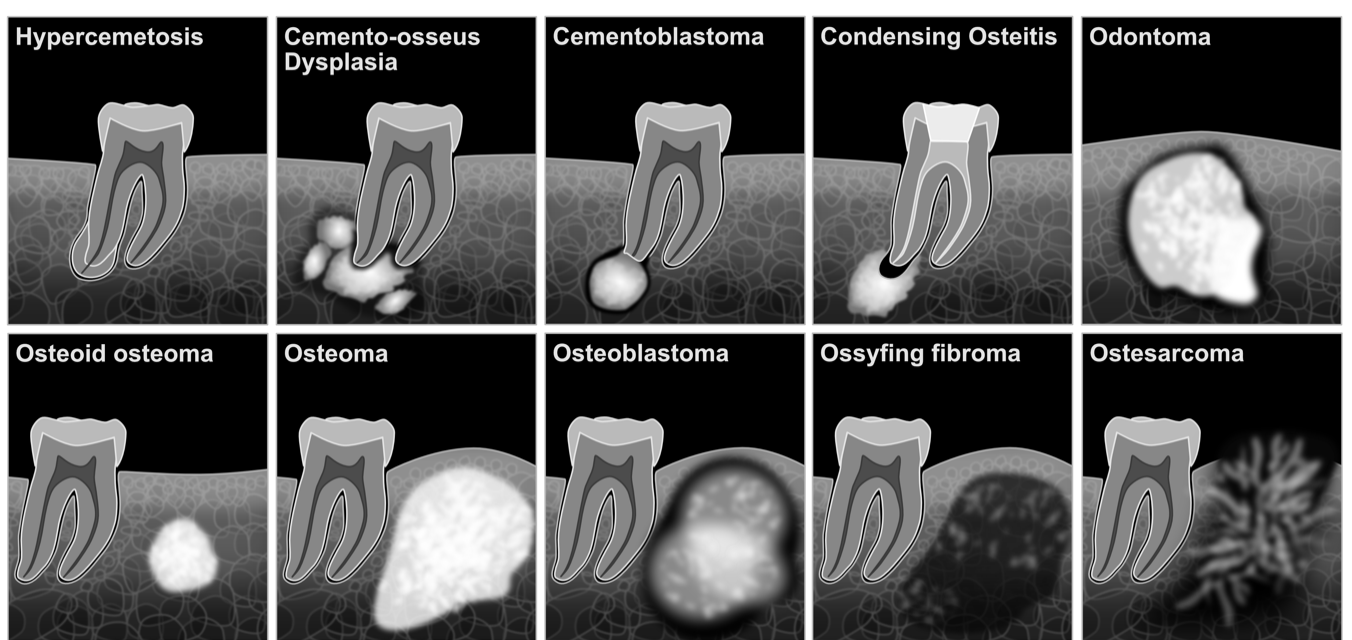
Schematic illustration of jaw bone lesions.

Cement-osseous dysplasia can be differentiated from osteomas radiographically because it varies from completely radiolucent to radiopaque mass. Furthermore, the average bone is replaced by a connective tissue matrix. Histologically, it is an avascular fibrous stroma with osteoid and some basophilic cemented structures in the early stage. Later, there is a more pronounced formation of osteoid trabeculae with the appearance of thicker curvilinear bony trabeculae and the appearance of prominent cemented masses [37]. In Figure 1 and Table A1, the characteristics of the mentioned bone lesions are illustrated and briefly summarized.

#### 3.1.3. Clinical Cases

A case of dental implantation in a woman with a central osteoma located in the left premolar region was reported by Carini et al. [6]. The woman came to the clinic to replace a compromised deciduous first molar. During radiographic examination, a well-circumscribed radiopaque mass without a radiolucent rim around and close to the roots of the teeth from the left maxillary canine to the third molar was revealed as an asymptomatic bony expansion. This mass affected the entire first impacted premolar. During the first surgery, the first impacted premolar was removed. At the same time, an excisional biopsy was performed. The biopsy showed the mature lamellar bone with no inflammation and cartilaginous tissue. A compact central osteoma was diagnosed. After four years of complete healing with dense and compact bone in the first premolar region formation, the second computed tomography revealed no major osteoma expansion. Therefore, the deciduous first premolar was removed in the second surgery, and the 3.6 × 13 mm implant (TSA Advance, DEFCON Tissue Care) was placed. After four months, a panoramic radiograph showed proper osseointegration and direct contact between the compact bone and the entire surface of the implant. At the same time, the provisional crown was replaced with a crown restoration [6].

Another report illustrated the potential for the dental implant to be installed in a woman with Gardner syndrome. Gardner’s syndrome is a hereditary autosomal dominant disease characterized by cysts of the sebaceous glands, osteomas, odontomas, and supernumerary teeth. The dental implant was placed in the upper jaw, and the osteoid osteoma area was functionally stable for a seven-year follow-up period [9].

The benign nature, low recurrence rate, and absence of inflammation signs provide a low probability of implant failure in the area of osteoid osteoma.

### 3.2. Odontoma

Odontoma is the most common odontogenic tumor [38]. It is benign and originates from an alteration of differentiated mesenchymal and epithelial odontogenic cells. Local traumatism, infection, Malassez paradental remains, odontoblastic hyperactivity, or hereditary abnormalities (Gardner and Herman syndrome) can be considered as potential etiological factors [27,39]. There is a predilection for the formation of odontomas in the incisor and canine area of the upper jaw, the lower anterior, and lower posterior areas of the lower jaw [38]. Clinically, two types of odontomas may be differentiated based on their location. Compound odontomas are usually located in the anterior maxilla, over the crowns of unerupted teeth, or between the roots of erupted teeth. Complex odontomas occur in the posterior mandible, often over an impacted tooth [40].

#### 3.2.1. Histopathologic Features

The compound odontoma is presented by dental tissue, including demineralized enamel, dentin, cement, and pulp. They organized the dental structures in an organized manner and were partially surrounded by a connective tissue capsule. Complex odontoma is a disorganized mass of hard dental tissues. Odontogenic and ameloblastic epithelium, phantom cells, and cementicles can be detected [41,42].

#### 3.2.2. Radiographic Features and Differential Diagnosis

Radiographically, compound odontomas can be observed as radiopaque masses with irregular margins that adopt a tooth-like configuration and radiolucid peripheral borders. Complex odontoma is a radioopaque mass which does not resemble tooth structure [43,44].

The odontoma could be differentiated from osteoma, ameloblastic fibro-odontoma, calcifying cystic odontogenic tumor, fibrous dysplasia, chronic osteomyelitis, cementoma, adenomatoid odontogenic tumor, supernumerary tooth, cementing fibroma, or benign osteoblastoma [38,41,42].

#### 3.2.3. Clinical Cases

The immediate dental implant placement after removal of the complex odontoma was performed in a 35-year-old woman. The surgical removal of the lesion was performed, and the implant was inserted. Inorganic bovine bone and bovine collagen membrane was used to fill the surgical cavity and suture was performed. The collected tissues were histopathologically analyzed, and the diagnosis of odontoma was confirmed. After 1 year, the implant was stable, and no complications were observed [45].

Successful dental rehabilitation of patients with complex odontoma were described [46,47]. To restore large bone defects after odontoma removal, a reconstruction plate without any bone grafting or anterior iliac crest graft was used. Histopathological examination was performed in both cases and confirmed the initial diagnosis of complex odontoma. Seven and 9 months later, a prosthetic rehabilitation was performed by placing dental implants.

Also cases of odontoma removal and delayed implant placement were presented. During the first surgical operation, the odontoma was removed. Implant placement was performed after 2 years and 9 months subsequently. After a 3-year follow-up, no signs of recurrence or complications were observed at the surgical site [48,49].

A review of the literature showed that removal of odontomas and immediate or delayed dental implantation could be successful and not accompanied by complications.

### 3.3. Osteoblastoma

Osteoblastoma is a benign tumor of osteoblastic origin. The etiologic factors are inflammation, trauma, and changes in bone physiology due to injury. Symptoms may include pain, swelling, and tenderness that may or may not be alleviated by nonsteroidal drugs. The size of the lesion was greater than 2 cm. There is a predilection for osteoblastoma formation in the posterior regions of mandible [27]. Osteoblastoma can be classified into two major clinicopathological forms. The benign form, which has a slow growth rate and a well-defined sclerotic margin, is moderately well vascularized with a mild inflammatory response. The aggressive form exhibits locally aggressive behavior with a tendency to recur, often complicating its differentiation from low-grade osteosarcoma.

#### 3.3.1. Histopathologic Features

Histopathological features of osteoblastoma include the presence of irregular bony trabecules, an outstanding vascular network and immature bone within the stroma. Bony trabeculae have various degrees of calcification and include several layers of plump hyperchromatic osteoblasts. Stromal cells are small and slender. In addition, in the area of the lesion, giant cells that are osteoblast-like and multinucleated can be observed. Moreover, remodeling of the osseous tissue might be seen in the form of basophilic reversal lines.

#### 3.3.2. Radiographic Features and Differential Diagnosis

Radiographically, osteoblastoma represents a well- or poorly-defined round to oval calcified area with or without radiolucency. Surrounding reactive sclerosis is less prominent in comparison with osteoid osteoma [27,50,51].

Osteoblastoma could be differentiated from other bone lesions, such as osteoid osteoma, cementoblastoma, fibrous dysplasia, cementosseous dysplasia, ossifying fibroma, and osteosarcoma.

In the literature, we have not found cases of dental implantation in the area of osteoblastoma. In our point of view, it is related to the possibility of inflammation, aggressive growth, and tendency to recurrence. In addition, en bloc resection is the treatment of choice for this lesion, which limits the possibility of dental implantation in the affected area.

### 3.4. Cementoblastoma

Cementoblastoma is a rare, slow-growing benign odontogenic tumor of mesenchymal origin. There is a preference for the mandible rather than for the maxilla. It is revealed mainly in the area of the molars [50,51]. In two-thirds of cases, cementoblastoma causes pain and swelling on the buccal and lingual sides due to expansion of the alveolar ridge [27]. The recurrence rate ranges from 5.9 to 37.1% [52,53].

#### 3.4.1. Histopathologic Features

Histopathological evidence is similar to those of osteoblastoma. A dense mass of cementoblasts is observed that includes mineralized cementum-like material with numerous basophilic reversal lines continuously fused with the tooth root. Multinucleated giant cells and plump cementoblasts may be present within the highly vascularized stroma [54]. There is no calcification of cemental material at the periphery, which is often arranged, perpendicular to the capsule.

#### 3.4.2. Radiographic Features and Differential Diagnosis

Radiographically, cementoblastoma manifests itself as a well-defined radiopaque lesion surrounded by a radiolucent zone. It attaches to the root of the tooth/teeth and resulting in resorption, loss of outline, and obliteration of the periodontal ligament space.

The differential diagnosis of cementoblastoma includes osteoblastoma, osteosarcoma, focal sclerosing osteomyelitis, hypercementosis, osteoid osteoma, odontoma, focal cement osseous dysplasia (FCOD), and condensing osteitis. The characteristics of the lesions have already been shown in Figure 1 and Table A1.

#### 3.4.3. Clinical Cases

In the literature, immediate implantation after removal of cementoblastoma was reported. A male patient complained of the wound presence in the mucosa after removing a remaining dental root 4 months earlier. During the radiographic examination, a well-defined radiopaque area was shown to be associated with the remaining root 4.6. The treatment plan based on the patient’s condition consisted of removing the tooth and tumor, immediate implant installation, and prosthodontic rehabilitation. After one year, there was no sign of implant mobility or inflammation [55].

There is also a study on delayed implantation. A 65-year-old patient came to the office with a complaint of facial asymmetry and moderate pain on the right side of the mandible body (symptoms of cortical expansion). Radiographic investigation showed a 10 mm radiaopaque lesion around the 4.6 tooth. The pulp vitality test for tooth 4.6 was positive. After excisional biopsy of the lesion and extraction of teeth 4.6 and 4.7, platelet-rich fibrin clots were put into the surgical site for healing. The result of biopsy showed it was cementoblastoma. Four months later, with no recurrent evidence, two 0.4 × 10 mm Neobiotech (R) implants were placed in the right mandible region and rehabilitated with an implant-supported bridge [56].

Cementoblastoma arises from cementoblasts and is attached to the root. After tooth extraction, the lesion and its remnants will also be removed. Implant placement can be done immediately or delayed after extraction of the lesion and corresponding tooth/teeth.

### 3.5. Cemento-Osseous Dysplasia (COD)

Cemento-osseous dysplasia is probably the most common fibro-osseous lesion that replaces normal bone with fibrous tissue with a newly formed mineralized component. The lesion comes from undifferentiated cells in the tissues of the periodontal ligament [57]. There are three types of COD: focal, periapical, and florid. Periapical cemento-osseous dysplasia predominantly manifests in the periapical area of vital anterior mandibular teeth in response to local factors and occurs adjacent to a tooth-bearing area as single or multiple lesions [27]. In this lesion, the dysplastic process is observed more than the neoplastic one. Focal cemento-osseous dysplasia is a single asymptomatic lesion prevalent in the posterior region of the mandible near the root or even in the edentulous area [58]. Florid cemento-osseous dysplasia is presented bilaterally and may occur symmetrically or affect all quadrants.

#### 3.5.1. Histopathologic Features

Histopathologically, the fibrovascular connective tissue is present with a mixture of woven structures, lamellar bone, and cementum-shaped structures. Later, the thickness of the bony trabeculae increases, and they become thick and curvilinear with shapes similar to those of the ginger roots. In the final stage, individual trabeculae fuse and form sheet-like or globular masses [27].

#### 3.5.2. Radiographical Investigation and Differential Diagnosis

In general, radiographical findings demonstrate the progress of COD from a predominantly radiolucent and mixed radiolucent to a predominantly radiopaque lesion in mature conditions. In the mixed and mature stages, the COD area is separated from the surrounding healthy bone by a radiolucent border without evidence of fusion to the tooth root [49].

Accurate differential diagnosis is obtained by considering the stage of COD progression. COD is differentiated from chronic osteomyelitis, ossifying fibroma, periapical granuloma, cyst, or periodontitis [22]. In mature form, it could differentiate from odontoma, cementoblastoma, osteoblastoma, and focal sclerosing osteomyelitis. Furthermore, in the case of florid cementoosseous dysplasia, the differential diagnosis includes chronic diffuse sclerosing osteomyelitis and ossifying fibroma. Serum alkaline phosphate (ALP) is assessed to differentiate florid cemento-osseous dysplasia from Paget’s disease: in Paget disease, ALP increases, whereas it is normal in florid cemento-osseous dysplasia.

#### 3.5.3. Clinical Cases

The osseointegration of the dental implant in the FCOD area was demonstrated in a 44-year-old woman. After panoramic X-ray, some irregular lobular and symmetric radiopacities surrounded by a radiolucent zone in the incisor, premolar, and molar areas on both sides of the mandible were discovered. In the mandibular first and second area, two implants were placed. After one month, the implants were restored with cement-retained metal-ceramic splinted crowns, and the patient was recommended to regular follow-up. After 8 years, follow-up orthopantomogram and periapical images showed optimal osseointegration and evaluation of soft tissues, including covering mucosa, presented no remarkable problem [59].

Implant survival during 15 years was shown by Park et al. (2019). However, at 16-years, the implant was removed due to periimplantitis and the cementum-like tissue attached to the implant was found. The authors recommend implant placement only after treatment of endodontic and periodontal infection and inflammation. In addition, because of the presence of immature tissue, implants should be placed into a late-stage FCOD lesion [60].

Implant rehabilitation was performed on a 48-year-old female. In the area of tooth 47, a radiopaque lobular lesion was found in orthopantomogram (OPG). Contribution of cone beam computed tomography (CBCT) in coronal view and OPG confirmed the lesion was focal cement-osseous dysplasia. Implant placement was done by following the three-stage protocol. The first stage is the drilling sequence with abundant cooling solution, rinsing with betadine of the newly created socket, hermetic closure of the wound, and prescription of antibiotic therapy. The second stage is reopening of the site after 3 weeks at the time of the proliferation phase of the socket healing process and implant placement. In the third stage, after a 3-month healing period, the cover screw is replaced with the healing abutment. According to the author’s recommendation, prescription of systemic antibiotics prevents the risk of infection and necrosis of surrounding tissues caused by heat production through drilling into dysplastic tissue [61].

The implant placement was successful in a 62-year-old woman with florid cemento-osseous dysplasia in edentulous regions of the mandible and mandibular anterior teeth. Two dental implants were placed in regions 36 and 37. Complete osteointegration of implants was shown on the X-ray images after 18 months. There were also no changes in the size of the FCOD lesion [5].

Implant insertion in the FCOD area can be successful but, in some cases, late implant failure occurs. These adverse events associated with blood supply and bone density disturbance. The dental implant failure after 6 months in a 40-year-old woman with FCOD was described by Oliveira et al. (2014) [62]. The patient complained of implant failure without sign of inflammation. Panoramic radiograph showed the dental implant was placed in the area of tooth 25, which was surrounded by an inconspicuous radiolucency. OPG and CBCT images represented a roughly ovoid well-corticated radiolucent lesion showing moderate degrees of mineralization at the center. Moreover, any examinations, including periapical radiographs before the time of implant installation, were not available for evaluation of doctor, which showed whether the mineralized area was presented before implantation or not. Furthermore, after incisional biopsy, they found that the mineralized area was attributable to FOCD, as detected in the second premolar area in both the right and left mandibular quadrants [62]. Preventing overheating during the drilling sequence for the installation of the dental implant in the dysplastic area is one of the most important criteria for avoiding implant failure. In fact, overheating lead to reduction of vascularization and capacity for bone regeneration due to necrosis of surrounding area and infection [61]. Furthermore, Shin et al. reported a case of failed osseointegration after dental implant placement in the area of cementoosseous dysplasia due to chronic osteomyelitis [63].

It has been illustrated that we are far from fully informed about the behavior of dysplastic bone with respect to dental implants. The probability of poor healing, infection, sequestrum formation, and fracture increases with surgical intervention regarding FCOD removal [17,64]. Therefore, some authors suggest that it is better to put the implant in case of FCOD without any procedure, which exposes the lesion. It will decrease the risk of implant failure [61].

### 3.6. Exostosis

Exostosis is a pathological bony outgrowth that gradually increase in size. It is often found in the lingual aspect of the mandible near the canine and premolar teeth (torus mandibularis) or uni- or bilaterally at the palatal midline [27,65]. The etiology of exostosis is unclear. Possible causes include genetic and environmental factors masticatory hyperfunction and continued bone growth. Several authors have postulated that the etiology is multifactorial, encompassing environmental and genetic occupational factors [27,66].

#### Histopathologic, Radiological Features, and Differential Diagnosis

Exostosis represents the hyperplasic bone, consisting of mature cortical and trabecular bone [22,27]. On CBCT exostoses are shown located in the inner aspect of the alveolar bone of the jaw above the origin of the mylohyoid muscle (mandibular tori), buccal cortex of the maxilla (buccal exostosis), the midline on the hard palate (torus palatinus).

The differential diagnosis of exostosis includes abscess formation, bone cancer, salivary gland tumors, vascular tumors, and fibromas.

Due to the location of exostoses, implantation in the area is not performed. But exostoses could be used as a source of autologous bone tissue to eliminate bone defects in nearby areas [67,68].

### 3.7. Idiopathic Osteosclerosis and Condensing Osteitis

Idiopathic osteosclerosis (IO) is the localized increase in compact bone tissue that develops in cancellous bone. It can be located in the apical regions of teeth, interradicular, or not be connected with the dentition. Also, idiopathic osteosclerosis does not appear to be associated with non-vital teeth. The lesion has a round, elliptical, or irregular shape and is usually a few millimeters to 1 to 3 cm in diameter. However, in some cases they can reach a size of 7 cm [69]. IO has an unknown etiology, but minor inflammation, such as occlusal trauma, during tooth replacement can cause it. Moreover, there was a hypothesis that IO may be a developmental anatomic variation of normal bone [70]. Longitudinal studies in adults show that IO remained stable or even decreased in size [70,71].

Condensing osteitis (chronic focal sclerosis osteomyelitis) is the radiopaque lesion present primarily in the posterior regions of the mandible around a root apex. The size of this lesion is usually between 2 and 6.5 mm [72]. CO results from a reaction to a dental-related infection or exposure to substances, such as arsenic trioxide (ATO), used for pulp devitaliation. Arsenic leaks through the apex and even the accessory canals of the roots and leads to necrosis of surrounding soft and supportive periodontal tissues or causes uneven bone remodeling by an initial increase in bone resorption followed by excessive bone formation osteoblast accumulation [73,74,75].

#### 3.7.1. Histopathologic Features

Due to a few indications for the removal of CO and IO, there is a little data on their histopathological features. Histological examination of condensing osteitis in cadaver species showed that CO represents the replacement of cancellous bone and marrow with compact bone. In some regions, fibrosis and an inflammatory infiltrate were seen but not in all specimens. [76]. IO have a scant fibrofatty marrow with dense lamellar bone and the presence or absence of inflammatory cells [27].

#### 3.7.2. Radiographic Features and Differential Diagnosis

In radiographic examination IO is shown as a well-defined round or irregular lesion [77]. It is sclerotic and has sharp margins. In 20% of patients, it is not related to a tooth. It is also described as a mandibular enostoses with elliptical shape without bone expansion [78]. Condensing osteitis on radiographs was presented as a periapical, poorly marginated, nonexpansile lesion associated with a nonvital tooth [79]. The difference between IO and CO has been discussed in different views. IO is presented by well-defined border with non-clear expansion to the adjuacent bone [78], while in another study, it is mentioned that IO in the maxillomandibular area is visible by well-defined border, but in 20% of cases IO has ill-defined borders [80]. In addition to each other, lesions should be differentiated from condensing osteitis, osteoma, osteoblastoma, cementoblastoma, and FCOD.

#### 3.7.3. Clinical Cases

Dental implantation in CO was reported in a patient who presented to the dental office with radiopaque mass attached to the root apex of tooth 45. After definitive diagnosis of CO based on radiologic and clinical investigations, tooth 45 was extracted. Immediately after extraction, guided bone regeneration was performed. After 6 months, dental implant was installed on the site [16].

Studies of implant stability after direct or delayed implantation in the IO area after removal of the lesion showed that both implants and peri-implant tissue were stable at one and two years of follow-up [7,18]. The authors write that there is no standard treatment protocol or treatment plan in cases of dental implantation in the area of IO. However, according to literature, if the IO have small size and apical position, the doctor should adjust the length, position and angulation of implant. When a large lesion is located coronally, direct implant placement might be performed. However, if the lesion has extremely high density, it can be removed, and delayed implant placement might be performed.

## 4. Discussion

The efficiency and safety of dental implantation in the area of mineralized jaw lesions is currently being discussed. A small number of reported cases can be associated with difficulties in diagnosing and avoiding complications related to a compromising reaction of bone and surrounding tissues [37,81]. In this regard, only a complete examination allows an accurate diagnosis and the choice of surgical tactics. Careful history can identify systemic diseases, trauma, pulpal inflammation, and arsenic exposure of adjacent or missing teeth in the area of injury that causes the appearance of hyperdense lesions. CBCT allows for detecting lesions with high accuracy and distinguishes with normal anatomical structures and artifacts compared to periapical and panoramic radiography. It can show the multidimensional structure of the lesion and its boundaries and changes in the size or shape of the lesion during dynamic observation [79,82]. Histopathological examination is the most informative method of neoplasms diagnostics. It allows to assess the predominance of osteoplastic, osteolytic, or neoplastic processes, differentiating hyperdense lesions from each other, and inflammatory and non-inflammatory jaw disease. The results of the clinical and radiographic examination may not coincide with the histological evidence, but this method can confirm or refute the diagnosis and choose treatment tactics [83,84].

All dental implantations, including successful ones, were performed using a standard sterile protocol with minimization of periosteal reflection, short procedure time, and avoidance of bone overheating and torque value corresponding to the implant manufacturers’ recommendations. Depending on the clinical situation, implant insertion could be performed directly in the area of the lesion, immediately after its removal, or delayed. The use of 2-stage protocol made it possible to prevent bone overheating when drilling mineralized bone tissues, as well as to assess the possibility of recurrence of the lesion and create conditions for the sufficient bone volume formation.

Regarding the effectiveness of dental implantation, the data from the literature review show that dental implantation could be successful in the area of osteoid osteoma, cementoosseous dysplasia of the odontoma, cementoma, condensing osteitis, and idiopathic osteosclerosis [30,50,74]. Inflammation, aggressive growth, and a tendency to recurrence are factors that limit dental implantation in the osteoblastoma area [7,8,27,48].

However, the obtained results have some limitations. There are few articles about dental implantation in hyperdense lesions. In our opinion, it can be related to that most of the cases of implantation in the area of jaw bone lesions could be accompanied by intra- and postoperative problems. Concerning this, doctors do not want to publish negative results for obvious reasons. Additionally, the clinical conditions and follow-up period duration in presented cases were different. It is also limiting us in deductions.

Thus, the problem of determining the effectiveness and safety of implantation in the area of hyperdense jaw lesions requires an additional systematic research. However, doctors should not avoid patients with such lesions. Before planning dental implantation, surgeons should pay attention to the diagnostic criteria: etiological factors, presence of clinical symptoms, radiological features, including size, nature of the lesion, location in the jaw, and relation to the teeth. Collecting clinical and radiological data can help make a preliminary diagnosis. Histopathological examination can confirm or refute diagnosis and determine further tactics. After surgery, regular and long-term clinical and radiological observations should be made for not less than 2 years [17,61].

## 5. Conclusions

Little research has been done on dental implant placement in patients with hyperdense lesions, but review of the literature allows us to conclude that implant placement could be performed successfully in the area of osteoid osteoma, condensing osteitis, and idiopathic osteosclerosis without removing the lesion. In the case of cementoblastoma, it is necessary to remove the causative tooth and the lesion and then perform a delayed or immediate implantation. The possibility of complications and implant failure limits the use of dental implants in the area of osteoblastoma and cemento-osseous dysplasia. To make the diagnosis accurate, oral surgeons should take precisely the patient’s history, make radiographs, CT, and histopathological examinations. It helps differentiate hyperdense lesions from each other and inflammatory and noninflammatory jaw disease. In addition, before surgery, physicians must evaluate all risks of dental implantation and discuss them with the patient. If the patient has undergone dental implantation, regular and long-term clinical and radiological observations are required.

## Data Availability

Not applicable.

## Appendix A

**Table A1:** Hyperdense jaw lesions–etiology, sex, and anatomical site predilection, histopathological and radiographic characteristics.

Lesion	Decades of Life/Age	Etiology	Gender Predilection	Anatomical Sites	Radiographic Characteristics	Histopathological Characteristics
Osteoid Osteoma	2nd–3rd	Benign neoplastic lesion	No.	Angle of mandible	Less than 1.5 cm centrally radiopacity surrounds a well-circumscribed round to ovoid radiolucency or nidus with reactive surrounding sclerosis.	Centrally located lamellar trabeculae of cancellous bone with ample fibrofatty bone marrow, which are surrounded by osteoblasts and scattered osteoclasts. Or the dense, compact bone with sparse marrow tissue, well-circumscribed highly vascularized nidus contains mixture of trabeculae of variably mineralized woven bone which surround central radiopacity.
Cementoblastoma	2nd–3rd	Benign neoplastic lesion	No.	Mostly in the area of the first molar of the mandible	Symptomatic round radiopaque mass with radiolucent rim. It is fused to the root of the tooth/teeth. May cause root resorption.	Dense mass of mineralized cementum-like material with numerous basophilic reversal lines.
Fibrous dysplasia	1st–2nd	Non-inflammatory	No.	Maxilla	The lesion ranges from a radiolucent to an entirely radiopaque lesion with a ground glass appearance.	Irregular trabeculae of immature bone with a slight to moderate cellular fibrous connective tissue stoma.
Ossifying fibroma	3rd–4th	Benign neoplasmic lesion	Female	Mandible in the area of molars and premolars	Asymptomatic, well-defined unilocular lesion with radiolucency or mixed radiolucency and radiopacity.	Cellular fibrous tissue with a mixture of cementicles, osteoid, and woven bone.
Osteosarcoma	2nd	Malignant bone tumor	Male	Maxilla and molar regions of the mandible	Symptomatic, mixed radiolucent radiopaque lesion, widened periodontal ligament and loss of periodontal space, destruction of cortical plate.	Infiltrative margins, cartilage formation, and presence of malignant cells without osteoid production.
Osteoblastoma	2nd–3rd	Benign neoplasm lesion	No.	Posterior area of the mandible	Asymptomatic, well-or–ill-defined round to oval calcified area with or without radiolucency or fully radiolucent.	Irregular bony trabeculae, outstanding vascular network, and immature bone within the stroma. Bony trabeculae show various degrees of calcification, several layers of plump, hyperchromatic osteoblasts.
Hypercementosis	2nd–3rd	Non-neoplasm excessive cementum deposition on the roots of teeth due to systemic and local factors	No.	Posterior region of the mandible	Asymptomatic, excessive dense mass around the root with irregular, surrounded by intact radiolucent periodontal ligament space and lamina dura.	Cellular or hypocellular excessive cementum.
Exostosis	5th	Benign protuberances of bone	No.	The lingual aspect of the mandible near the canine and premolar teeth (torus mandibularis) or uni- or bilaterally in the palatal midline (torus palatinus and buccal exostoses)	Hyperplasic bone, consisting of mature cortical and trabecular bone.	Bony outgrowths located in the inner aspect of the alveolar bone of the jaw above the origin of the mylohyoid muscle (mandibular tori), buccal cortex of the maxilla (buccal exostosis), the midline on the hard palate (torus palatinus).
Odontoma	1st–2nd	Odontogenic tumor-like malformation	No.	Incisor and canine areas of the maxilla and mandible	Amorphous radiopaque mass surrounded by slight radiolucency. Compound odontoma: radiopaque tooth structure in the tooth-bearing area, between roots, or over the crown of the impacted tooth.	Normal tooth component structures like enamel, dentine, cementum and even pulp, connective tissue capsules with islands of odontogenic epithelium.
Condensing osteitis (Focal sclerosing osteomyelitis)	3rd–7th	Low-grade inflammatory stimulus from an inflamed dental pulp	No.	In the molar and premolar area of the mandible and associated with infected teeth	No radiolucent border, poorly defined nonexpanding sclerotic image, thickening of the periodontal ligament space, diffuse radiopaque lesions, and may be combined with adjacent radiolucent.inflammatory lesions.	Replacement of bone marrow and cancellous bone with dense compact bone fibrosis replacing fatty marrow.
